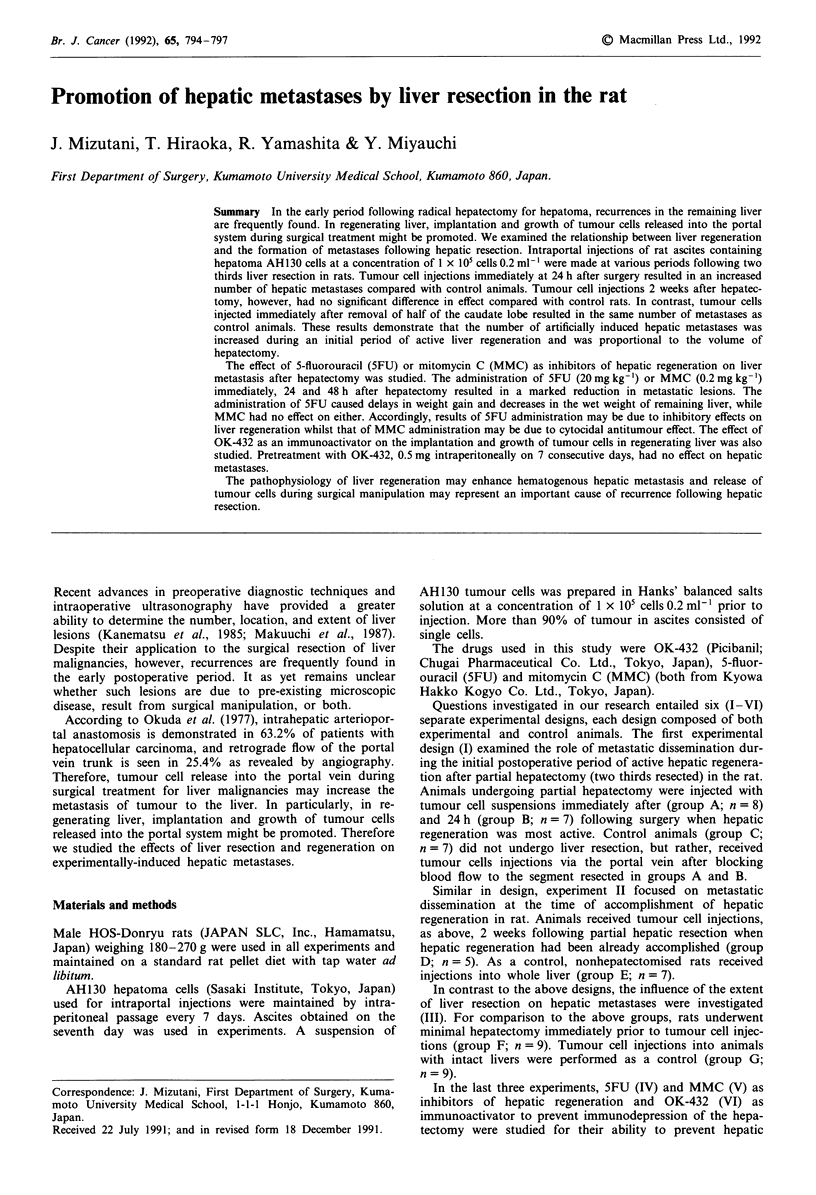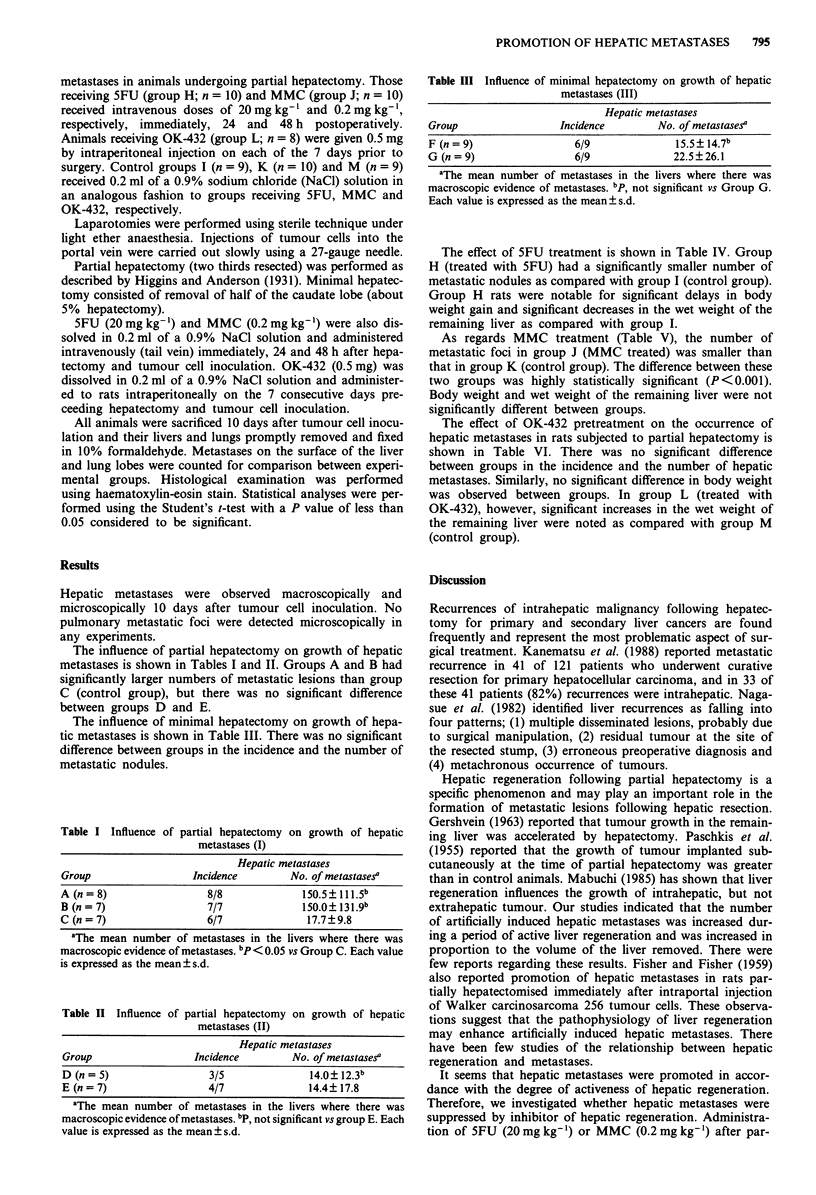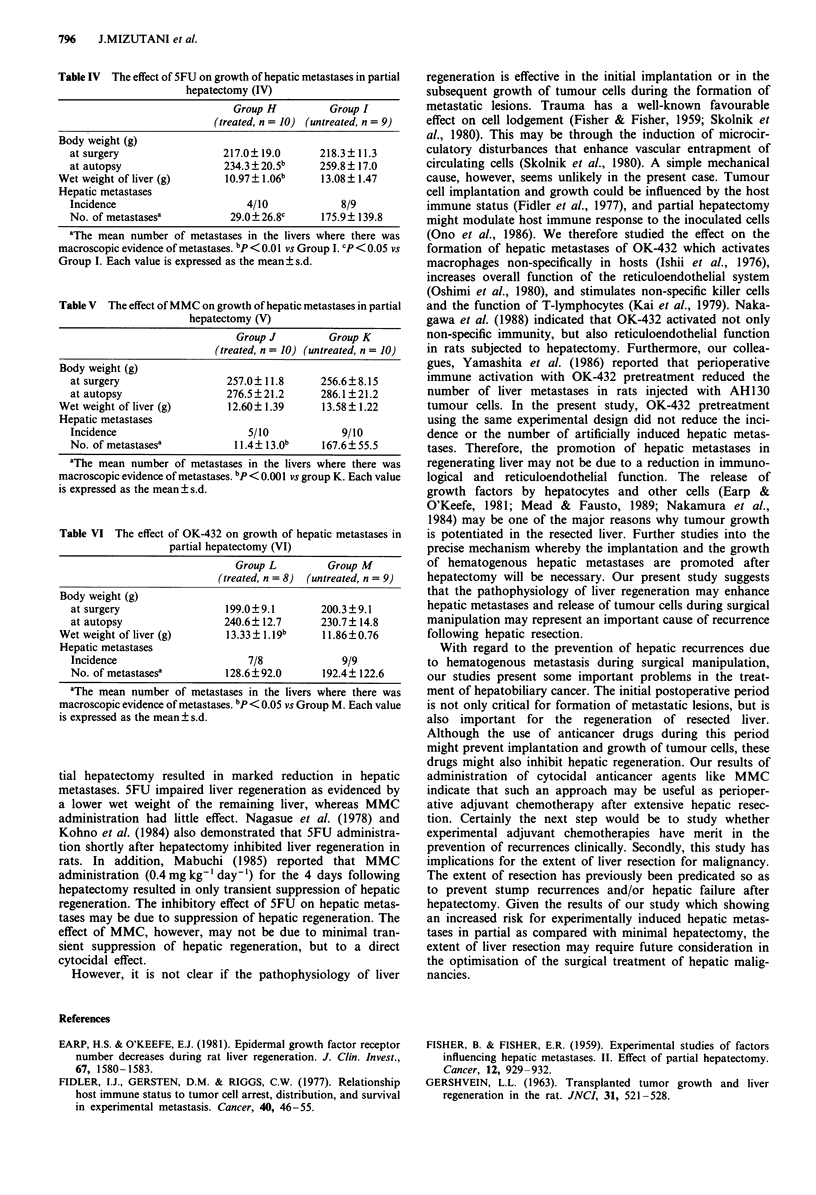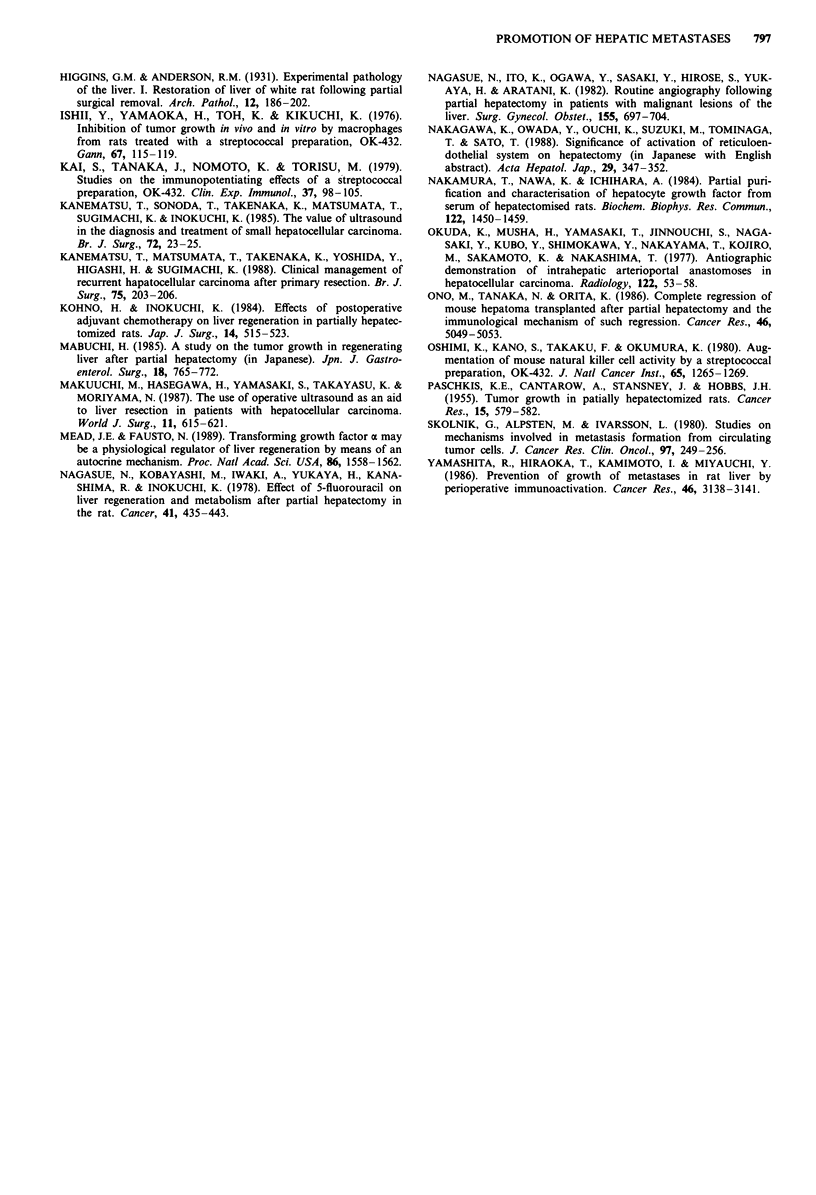# Promotion of hepatic metastases by liver resection in the rat.

**DOI:** 10.1038/bjc.1992.170

**Published:** 1992-06

**Authors:** J. Mizutani, T. Hiraoka, R. Yamashita, Y. Miyauchi

**Affiliations:** First Department of Surgery, Kumamoto University Medical School, Japan.

## Abstract

In the early period following radical hepatectomy for hepatoma, recurrences in the remaining liver are frequently found. In regenerating liver, implantation and growth of tumour cells released into the portal system during surgical treatment might be promoted. We examined the relationship between liver regeneration and the formation of metastases following hepatic resection. Intraportal injections of rat ascites containing hepatoma AH130 cells at a concentration of 1 x 10(5) cells 0.2 ml-1 were made at various periods following two thirds liver resection in rats. Tumour cell injections immediately at 24 h after surgery resulted in an increased number of hepatic metastases compared with control animals. Tumour cell injections 2 weeks after hepatectomy, however, had no significant difference in effect compared with control rats. In contrast, tumour cells injected immediately after removal of half of the caudate lobe resulted in the same number of metastases as control animals. These results demonstrate that the number of artificially induced hepatic metastases was increased during an initial period of active liver regeneration and was proportional to the volume of hepatectomy. The effect of 5-fluorouracil (5FU) or mitomycin C (MMC) as inhibitors of hepatic regeneration on liver metastasis after hepatectomy was studied. The administration of 5FU (20 mg kg-1) or MMC (0.2 mg kg-1) immediately, 24 and 48 h after hepatectomy resulted in a marked reduction in metastatic lesions. The administration of 5FU caused delays in weight gain and decreases in the wet weight of remaining liver, while MMC had no effect on either. Accordingly, results of 5FU administration may be due to inhibitory effects on liver regeneration whilst that of MMC administration may be due to cytocidal antitumour effect. The effect of OK-432 as an immunoactivator on the implantation and growth of tumour cells in regenerating liver was also studied. Pretreatment with OK-432, 0.5 mg intraperitoneally on 7 consecutive days, had no effect on hepatic metastases. The pathophysiology of liver regeneration may enhance hematogenous hepatic metastasis and release of tumour cells during surgical manipulation may represent an important cause of recurrence following hepatic resection.


Br. J. Cancer (1992), 65, 794-797 © Macmillan Press Ltd., 1992

### Promotion of hepatic metastases by liver resection in the rat

J. Mizutani, T. Hiraoka, R. Yamashita & Y. Miyauchi

*First Department of Surgery, Kumamoto University Medical School, Kumamoto 860, Japan.*

**Summary** In the early period following radical hepatectomy for hepatoma, recurrences in the remaining liver are frequently found. In regenerating liver, implantation and growth of tumour cells released into the portal system during surgical treatment might be promoted. We examined the relationship between liver regeneration and the formation of metastases following hepatic resection. Intraportal injections of rat ascites containing hepatoma AH130 cells at a concentration of $1 \times 10^5$ cells 0.2 ml$^{-1}$ were made at various periods following two thirds liver resection in rats. Tumour cell injections immediately at 24 h after surgery resulted in an increased number of hepatic metastases compared with control animals. Tumour cell injections 2 weeks after hepatectomy, however, had no significant difference in effect compared with control rats. In contrast, tumour cells injected immediately after removal of half of the caudate lobe resulted in the same number of metastases as control animals. These results demonstrate that the number of artificially induced hepatic metastases was increased during an initial period of active liver regeneration and was proportional to the volume of hepatectomy.

The effect of 5-fluorouracil (5FU) or mitomycin C (MMC) as inhibitors of hepatic regeneration on liver metastasis after hepatectomy was studied. The administration of 5FU (20 mg kg$^{-1}$) or MMC (0.2 mg kg$^{-1}$) immediately, 24 and 48 h after hepatectomy resulted in a marked reduction in metastatic lesions. The administration of 5FU caused delays in weight gain and decreases in the wet weight of remaining liver, while MMC had no effect on either. Accordingly, results of 5FU administration may be due to inhibitory effects on liver regeneration whilst that of MMC administration may be due to cytocidal antitumour effect. The effect of OK-432 as an immunoactivator on the implantation and growth of tumour cells in regenerating liver was also studied. Pretreatment with OK-432, 0.5 mg intraperitoneally on 7 consecutive days, had no effect on hepatic metastases.

The pathophysiology of liver regeneration may enhance hematogenous hepatic metastasis and release of tumour cells during surgical manipulation may represent an important cause of recurrence following hepatic resection.

Recent advances in preoperative diagnostic techniques and intraoperative ultrasonography have provided a greater ability to determine the number, location, and extent of liver lesions (Kanematsu *et al.*, 1985; Makuuchi *et al.*, 1987). Despite their application to the surgical resection of liver malignancies, however, recurrences are frequently found in the early postoperative period. It as yet remains unclear whether such lesions are due to pre-existing microscopic disease, result from surgical manipulation, or both.

According to Okuda *et al.* (1977), intrahepatic arterioportal anastomosis is demonstrated in 63.2% of patients with hepatocellular carcinoma, and retrograde flow of the portal vein trunk is seen in 25.4% as revealed by angiography. Therefore, tumour cell release into the portal vein during surgical treatment for liver malignancies may increase the metastasis of tumour to the liver. In particularly, in regenerating liver, implantation and growth of tumour cells released into the portal system might be promoted. Therefore we studied the effects of liver resection and regeneration on experimentally-induced hepatic metastases.

### Materials and methods

Male HOS-Donryu rats (JAPAN SLC, Inc., Hamamatsu, Japan) weighing 180-270 g were used in all experiments and maintained on a standard rat pellet diet with tap water *ad libitum*.

AH130 hepatoma cells (Sasaki Institute, Tokyo, Japan) used for intraportal injections were maintained by intraperitoneal passage every 7 days. Ascites obtained on the seventh day was used in experiments. A suspension of AH130 tumour cells was prepared in Hanks' balanced salts solution at a concentration of $1 \times 10^5$ cells 0.2 ml$^{-1}$ prior to injection. More than 90% of tumour in ascites consisted of single cells.

The drugs used in this study were OK-432 (Picibanil; Chugai Pharmaceutical Co. Ltd., Tokyo, Japan), 5-fluorouracil (5FU) and mitomycin C (MMC) (both from Kyowa Hakko Kogyo Co. Ltd., Tokyo, Japan).

Questions investigated in our research entailed six (I-VI) separate experimental designs, each design composed of both experimental and control animals. The first experimental design (I) examined the role of metastatic dissemination during the initial postoperative period of active hepatic regeneration after partial hepatectomy (two thirds resected) in the rat. Animals undergoing partial hepatectomy were injected with tumour cell suspensions immediately after (group A; $n = 8$) and 24 h (group B; $n = 7$) following surgery when hepatic regeneration was most active. Control animals (group C; $n = 7$) did not undergo liver resection, but rather, received tumour cells injections via the portal vein after blocking blood flow to the segment resected in groups A and B.

Similar in design, experiment II focused on metastatic dissemination at the time of accomplishment of hepatic regeneration in rat. Animals received tumour cell injections, as above, 2 weeks following partial hepatic resection when hepatic regeneration had been already accomplished (group D; $n = 5$). As a control, nonhepatectomised rats received injections into whole liver (group E; $n = 7$).

In contrast to the above designs, the influence of the extent of liver resection on hepatic metastases were investigated (III). For comparison to the above groups, rats underwent minimal hepatectomy immediately prior to tumour cell injections (group F; $n = 9$). Tumour cell injections into animals with intact livers were performed as a control (group G; $n = 9$).

In the last three experiments, 5FU (IV) and MMC (V) as inhibitors of hepatic regeneration and OK-432 (VI) as immunoactivator to prevent immunodepression of the hepatectomy were studied for their ability to prevent hepatic

Correspondence: J. Mizutani, First Department of Surgery, Kumamoto University Medical School, 1-1-1 Honjo, Kumamoto 860, Japan.
Received 22 July 1991; and in revised form 18 December 1991.

metastases in animals undergoing partial hepatectomy. Those receiving 5FU (group H; $n = 10$) and MMC (group J; $n = 10$) received intravenous doses of $20\ mg\ kg^{-1}$ and $0.2\ mg\ kg^{-1}$, respectively, immediately, 24 and 48 h postoperatively. Animals receiving OK-432 (group L; $n = 8$) were given 0.5 mg by intraperitoneal injection on each of the 7 days prior to surgery. Control groups I ($n = 9$), K ($n = 10$) and M ($n = 9$) received 0.2 ml of a 0.9% sodium chloride (NaCl) solution in an analogous fashion to groups receiving 5FU, MMC and OK-432, respectively.

Laparotomies were performed using sterile technique under light ether anaesthesia. Injections of tumour cells into the portal vein were carried out slowly using a 27-gauge needle.

Partial hepatectomy (two thirds resected) was performed as described by Higgins and Anderson (1931). Minimal hepatectomy consisted of removal of half of the caudate lobe (about 5% hepatectomy).

5FU ($20\ mg\ kg^{-1}$) and MMC ($0.2\ mg\ kg^{-1}$) were also dissolved in 0.2 ml of a 0.9% NaCl solution and administered intravenously (tail vein) immediately, 24 and 48 h after hepatectomy and tumour cell inoculation. OK-432 (0.5 mg) was dissolved in 0.2 ml of a 0.9% NaCl solution and administered to rats intraperitoneally on the 7 consecutive days preceeding hepatectomy and tumour cell inoculation.

All animals were sacrificed 10 days after tumour cell inoculation and their livers and lungs promptly removed and fixed in 10% formaldehyde. Metastases on the surface of the liver and lung lobes were counted for comparison between experimental groups. Histological examination was performed using haematoxylin-eosin stain. Statistical analyses were performed using the Student's $t$-test with a $P$ value of less than 0.05 considered to be significant.

### Results

Hepatic metastases were observed macroscopically and microscopically 10 days after tumour cell inoculation. No pulmonary metastatic foci were detected microscopically in any experiments.

The influence of partial hepatectomy on growth of hepatic metastases is shown in Tables I and II. Groups A and B had significantly larger numbers of metastatic lesions than group C (control group), but there was no significant difference between groups D and E.

The influence of minimal hepatectomy on growth of hepatic metastases is shown in Table III. There was no significant difference between groups in the incidence and the number of metastatic nodules.

**Table I** Influence of partial hepatectomy on growth of hepatic metastases (I)

| Group | Hepatic metastases Incidence | No. of metastases[a] |
|---|---|---|
| A ($n = 8$) | 8/8 | 150.5±111.5[b] |
| B ($n = 7$) | 7/7 | 150.0±131.9[b] |
| C ($n = 7$) | 6/7 | 17.7±9.8 |

[a]The mean number of metastases in the livers where there was macroscopic evidence of metastases. [b]$P < 0.05$ *vs* Group C. Each value is expressed as the mean ± s.d.

**Table II** Influence of partial hepatectomy on growth of hepatic metastases (II)

| Group | Hepatic metastases Incidence | No. of metastases[a] |
|---|---|---|
| D ($n = 5$) | 3/5 | 14.0±12.3[b] |
| E ($n = 7$) | 4/7 | 14.4±17.8 |

[a]The mean number of metastases in the livers where there was macroscopic evidence of metastases. [b]P, not significant *vs* group E. Each value is expressed as the mean ± s.d.

**Table III** Influence of minimal hepatectomy on growth of hepatic metastases (III)

| Group | Hepatic metastases Incidence | No. of metastases[a] |
|---|---|---|
| F ($n = 9$) | 6/9 | 15.5±14.7[b] |
| G ($n = 9$) | 6/9 | 22.5±26.1 |

[a]The mean number of metastases in the livers where there was macroscopic evidence of metastases. [b]$P$, not significant *vs* Group G. Each value is expressed as the mean ± s.d.

The effect of 5FU treatment is shown in Table IV. Group H (treated with 5FU) had a significantly smaller number of metastatic nodules as compared with group I (control group). Group H rats were notable for significant delays in body weight gain and significant decreases in the wet weight of the remaining liver as compared with group I.

As regards MMC treatment (Table V), the number of metastatic foci in group J (MMC treated) was smaller than that in group K (control group). The difference between these two groups was highly statistically significant ($P < 0.001$). Body weight and wet weight of the remaining liver were not significantly different between groups.

The effect of OK-432 pretreatment on the occurrence of hepatic metastases in rats subjected to partial hepatectomy is shown in Table VI. There was no significant difference between groups in the incidence and the number of hepatic metastases. Similarly, no significant difference in body weight was observed between groups. In group L (treated with OK-432), however, significant increases in the wet weight of the remaining liver were noted as compared with group M (control group).

### Discussion

Recurrences of intrahepatic malignancy following hepatectomy for primary and secondary liver cancers are found frequently and represent the most problematic aspect of surgical treatment. Kanematsu *et al.* (1988) reported metastatic recurrence in 41 of 121 patients who underwent curative resection for primary hepatocellular carcinoma, and in 33 of these 41 patients (82%) recurrences were intrahepatic. Nagasue *et al.* (1982) identified liver recurrences as falling into four patterns; (1) multiple disseminated lesions, probably due to surgical manipulation, (2) residual tumour at the site of the resected stump, (3) erroneous preoperative diagnosis and (4) metachronous occurrence of tumours.

Hepatic regeneration following partial hepatectomy is a specific phenomenon and may play an important role in the formation of metastatic lesions following hepatic resection. Gershvein (1963) reported that tumour growth in the remaining liver was accelerated by hepatectomy. Paschkis *et al.* (1955) reported that the growth of tumour implanted subcutaneously at the time of partial hepatectomy was greater than in control animals. Mabuchi (1985) has shown that liver regeneration influences the growth of intrahepatic, but not extrahepatic tumour. Our studies indicated that the number of artificially induced hepatic metastases was increased during a period of active liver regeneration and was increased in proportion to the volume of the liver removed. There were few reports regarding these results. Fisher and Fisher (1959) also reported promotion of hepatic metastases in rats partially hepatectomised immediately after intraportal injection of Walker carcinosarcoma 256 tumour cells. These observations suggest that the pathophysiology of liver regeneration may enhance artificially induced hepatic metastases. There have been few studies of the relationship between hepatic regeneration and metastases.

It seems that hepatic metastases were promoted in accordance with the degree of activeness of hepatic regeneration. Therefore, we investigated whether hepatic metastases were suppressed by inhibitor of hepatic regeneration. Administration of 5FU ($20\ mg\ kg^{-1}$) or MMC ($0.2\ mg\ kg^{-1}$) after par-

**Table IV** The effect of 5FU on growth of hepatic metastases in partial hepatectomy (IV)

| | Group H (treated, n = 10) | Group I (untreated, n = 9) |
|---|---|---|
| Body weight (g) | | |
| at surgery | 217.0±19.0 | 218.3±11.3 |
| at autopsy | 234.3±20.5[b] | 259.8±17.0 |
| Wet weight of liver (g) | 10.97±1.06[b] | 13.08±1.47 |
| Hepatic metastases | | |
| Incidence | 4/10 | 8/9 |
| No. of metastases[a] | 29.0±26.8[c] | 175.9±139.8 |

[a]The mean number of metastases in the livers where there was macroscopic evidence of metastases. [b]$P<0.01$ vs Group I. [c]$P<0.05$ vs Group I. Each value is expressed as the mean±s.d.

**Table V** The effect of MMC on growth of hepatic metastases in partial hepatectomy (V)

| | Group J (treated, n = 10) | Group K (untreated, n = 10) |
|---|---|---|
| Body weight (g) | | |
| at surgery | 257.0±11.8 | 256.6±8.15 |
| at autopsy | 276.5±21.2 | 286.1±21.2 |
| Wet weight of liver (g) | 12.60±1.39 | 13.58±1.22 |
| Hepatic metastases | | |
| Incidence | 5/10 | 9/10 |
| No. of metastases[a] | 11.4±13.0[b] | 167.6±55.5 |

[a]The mean number of metastases in the livers where there was macroscopic evidence of metastases. [b]$P<0.001$ vs group K. Each value is expressed as the mean±s.d.

**Table VI** The effect of OK-432 on growth of hepatic metastases in partial hepatectomy (VI)

| | Group L (treated, n = 8) | Group M (untreated, n = 9) |
|---|---|---|
| Body weight (g) | | |
| at surgery | 199.0±9.1 | 200.3±9.1 |
| at autopsy | 240.6±12.7 | 230.7±14.8 |
| Wet weight of liver (g) | 13.33±1.19[b] | 11.86±0.76 |
| Hepatic metastases | | |
| Incidence | 7/8 | 9/9 |
| No. of metastases[a] | 128.6±92.0 | 192.4±122.6 |

[a]The mean number of metastases in the livers where there was macroscopic evidence of metastases. [b]$P<0.05$ vs Group M. Each value is expressed as the mean±s.d.

tial hepatectomy resulted in marked reduction in hepatic metastases. 5FU impaired liver regeneration as evidenced by a lower wet weight of the remaining liver, whereas MMC administration had little effect. Nagasue *et al.* (1978) and Kohno *et al.* (1984) also demonstrated that 5FU administration shortly after hepatectomy inhibited liver regeneration in rats. In addition, Mabuchi (1985) reported that MMC administration (0.4 mg $kg^{-1}$ $day^{-1}$) for the 4 days following hepatectomy resulted in only transient suppression of hepatic regeneration. The inhibitory effect of 5FU on hepatic metastases may be due to suppression of hepatic regeneration. The effect of MMC, however, may not be due to minimal transient suppression of hepatic regeneration, but to a direct cytocidal effect.

However, it is not clear if the pathophysiology of liver regeneration is effective in the initial implantation or in the subsequent growth of tumour cells during the formation of metastatic lesions. Trauma has a well-known favourable effect on cell lodgement (Fisher & Fisher, 1959; Skolnik *et al.*, 1980). This may be through the induction of microcirculatory disturbances that enhance vascular entrapment of circulating cells (Skolnik *et al.*, 1980). A simple mechanical cause, however, seems unlikely in the present case. Tumour cell implantation and growth could be influenced by the host immune status (Fidler *et al.*, 1977), and partial hepatectomy might modulate host immune response to the inoculated cells (Ono *et al.*, 1986). We therefore studied the effect on the formation of hepatic metastases of OK-432 which activates macrophages non-specifically in hosts (Ishii *et al.*, 1976), increases overall function of the reticuloendothelial system (Oshimi *et al.*, 1980), and stimulates non-specific killer cells and the function of T-lymphocytes (Kai *et al.*, 1979). Nakagawa *et al.* (1988) indicated that OK-432 activated not only non-specific immunity, but also reticuloendothelial function in rats subjected to hepatectomy. Furthermore, our colleagues, Yamashita *et al.* (1986) reported that perioperative immune activation with OK-432 pretreatment reduced the number of liver metastases in rats injected with AH130 tumour cells. In the present study, OK-432 pretreatment using the same experimental design did not reduce the incidence or the number of artificially induced hepatic metastases. Therefore, the promotion of hepatic metastases in regenerating liver may not be due to a reduction in immunological and reticuloendothelial function. The release of growth factors by hepatocytes and other cells (Earp & O'Keefe, 1981; Mead & Fausto, 1989; Nakamura *et al.*, 1984) may be one of the major reasons why tumour growth is potentiated in the resected liver. Further studies into the precise mechanism whereby the implantation and the growth of hematogenous hepatic metastases are promoted after hepatectomy will be necessary. Our present study suggests that the pathophysiology of liver regeneration may enhance hepatic metastases and release of tumour cells during surgical manipulation may represent an important cause of recurrence following hepatic resection.

With regard to the prevention of hepatic recurrences due to hematogenous metastasis during surgical manipulation, our studies present some important problems in the treatment of hepatobiliary cancer. The initial postoperative period is not only critical for formation of metastatic lesions, but is also important for the regeneration of resected liver. Although the use of anticancer drugs during this period might prevent implantation and growth of tumour cells, these drugs might also inhibit hepatic regeneration. Our results of administration of cytocidal anticancer agents like MMC indicate that such an approach may be useful as perioperative adjuvant chemotherapy after extensive hepatic resection. Certainly the next step would be to study whether experimental adjuvant chemotherapies have merit in the prevention of recurrences clinically. Secondly, this study has implications for the extent of liver resection for malignancy. The extent of resection has previously been predicated so as to prevent stump recurrences and/or hepatic failure after hepatectomy. Given the results of our study which showing an increased risk for experimentally induced hepatic metastases in partial as compared with minimal hepatectomy, the extent of liver resection may require future consideration in the optimisation of the surgical treatment of hepatic malignancies.

### References

EARP, H.S. & O'KEEFE, E.J. (1981). Epidermal growth factor receptor number decreases during rat liver regeneration. *J. Clin. Invest.*, **67**, 1580–1583.

FIDLER, I.J., GERSTEN, D.M. & RIGGS, C.W. (1977). Relationship host immune status to tumor cell arrest, distribution, and survival in experimental metastasis. *Cancer*, **40**, 46–55.

FISHER, B. & FISHER, E.R. (1959). Experimental studies of factors influencing hepatic metastases. II. Effect of partial hepatectomy. *Cancer*, **12**, 929–932.

GERSHVEIN, L.L. (1963). Transplanted tumor growth and liver regeneration in the rat. *JNCI*, **31**, 521–528.

HIGGINS, G.M. & ANDERSON, R.M. (1931). Experimental pathology of the liver. I. Restoration of liver of white rat following partial surgical removal. *Arch. Pathol.*, **12,** 186–202.

ISHII, Y., YAMAOKA, H., TOH, K. & KIKUCHI, K. (1976). Inhibition of tumor growth *in vivo* and *in vitro* by macrophages from rats treated with a streptococcal preparation, OK-432. *Gann*, **67,** 115–119.

KAI, S., TANAKA, J., NOMOTO, K. & TORISU, M. (1979). Studies on the immunopotentiating effects of a streptococcal preparation, OK-432. *Clin. Exp. Immunol.*, **37,** 98–105.

KANEMATSU, T., SONODA, T., TAKENAKA, K., MATSUMATA, T., SUGIMACHI, K. & INOKUCHI, K. (1985). The value of ultrasound in the diagnosis and treatment of small hepatocellular carcinoma. *Br. J. Surg.*, **72,** 23–25.

KANEMATSU, T., MATSUMATA, T., TAKENAKA, K., YOSHIDA, Y., HIGASHI, H. & SUGIMACHI, K. (1988). Clinical management of recurrent hapatocellular carcinoma after primary resection. *Br. J. Surg.*, **75,** 203–206.

KOHNO, H. & INOKUCHI, K. (1984). Effects of postoperative adjuvant chemotherapy on liver regeneration in partially hepatectomized rats. *Jap. J. Surg.*, **14,** 515–523.

MABUCHI, H. (1985). A study on the tumor growth in regenerating liver after partial hepatectomy (in Japanese). *Jpn. J. Gastroenterol. Surg.*, **18,** 765–772.

MAKUUCHI, M., HASEGAWA, H., YAMASAKI, S., TAKAYASU, K. & MORIYAMA, N. (1987). The use of operative ultrasound as an aid to liver resection in patients with hepatocellular carcinoma. *World J. Surg.*, **11,** 615–621.

MEAD, J.E. & FAUSTO, N. (1989). Transforming growth factor α may be a physiological regulator of liver regeneration by means of an autocrine mechanism. *Proc. Natl Acad. Sci. USA*, **86,** 1558–1562.

NAGASUE, N., KOBAYASHI, M., IWAKI, A., YUKAYA, H., KANASHIMA, R. & INOKUCHI, K. (1978). Effect of 5-fluorouracil on liver regeneration and metabolism after partial hepatectomy in the rat. *Cancer*, **41,** 435–443.

NAGASUE, N., ITO, K., OGAWA, Y., SASAKI, Y., HIROSE, S., YUKAYA, H. & ARATANI, K. (1982). Routine angiography following partial hepatectomy in patients with malignant lesions of the liver. *Surg. Gynecol. Obstet.*, **155,** 697–704.

NAKAGAWA, K., OWADA, Y., OUCHI, K., SUZUKI, M., TOMINAGA, T. & SATO, T. (1988). Significance of activation of reticuloendothelial system on hepatectomy (in Japanese with English abstract). *Acta Hepatol. Jap.*, **29,** 347–352.

NAKAMURA, T., NAWA, K. & ICHIHARA, A. (1984). Partial purification and characterisation of hepatocyte growth factor from serum of hepatectomised rats. *Biochem. Biophys. Res. Commun.*, **122,** 1450–1459.

OKUDA, K., MUSHA, H., YAMASAKI, T., JINNOUCHI, S., NAGASAKI, Y., KUBO, Y., SHIMOKAWA, Y., NAKAYAMA, T., KOJIRO, M., SAKAMOTO, K. & NAKASHIMA, T. (1977). Antiographic demonstration of intrahepatic arterioportal anastomoses in hepatocellular carcinoma. *Radiology*, **122,** 53–58.

ONO, M., TANAKA, N. & ORITA, K. (1986). Complete regression of mouse hepatoma transplanted after partial hepatectomy and the immunological mechanism of such regression. *Cancer Res.*, **46,** 5049–5053.

OSHIMI, K., KANO, S., TAKAKU, F. & OKUMURA, K. (1980). Augmentation of mouse natural killer cell activity by a streptococcal preparation, OK-432. *J. Natl Cancer Inst.*, **65,** 1265–1269.

PASCHKIS, K.E., CANTAROW, A., STANSNEY, J. & HOBBS, J.H. (1955). Tumor growth in patially hepatectomized rats. *Cancer Res.*, **15,** 579–582.

SKOLNIK, G., ALPSTEN, M. & IVARSSON, L. (1980). Studies on mechanisms involved in metastasis formation from circulating tumor cells. *J. Cancer Res. Clin. Oncol.*, **97,** 249–256.

YAMASHITA, R., HIRAOKA, T., KAMIMOTO, I. & MIYAUCHI, Y. (1986). Prevention of growth of metastases in rat liver by perioperative immunoactivation. *Cancer Res.*, **46,** 3138–3141.